# Environmental Health Indicators of Climate Change for the United States: Findings from the State Environmental Health Indicator Collaborative

**DOI:** 10.1289/ehp.0900708

**Published:** 2009-05-18

**Authors:** Paul B. English, Amber H. Sinclair, Zev Ross, Henry Anderson, Vicki Boothe, Christine Davis, Kristie Ebi, Betsy Kagey, Kristen Malecki, Rebecca Shultz, Erin Simms

**Affiliations:** 1 Center for Chronic Disease Prevention and Health Promotion, California Department of Public Health, Richmond, California, USA; 2 University of Georgia, Department of Public Administration and Policy, Augusta, Georgia, USA; 3 Zev Ross Spatial Analysis, Ithaca, New York, USA; 4 Wisconsin Division of Public Health, Madison, Wisconsin, USA; 5 Coordinating Center for Environmental Health and Injury Prevention, U.S. Centers for Disease Control and Prevention, Atlanta, Georgia, USA; 6 Climate, International, and Multimedia Group, U.S. Environmental Protection Agency, Research Triangle Park, North Carolina, USA; 7 ESS, LLC, Alexandria, Virginia, USA; 8 Division of Public Health, Georgia Department of Human Resources, Atlanta, Georgia, USA; 9 Florida Department of Health, Tallahassee, Florida, USA; 10 Council of State and Territorial Epidemiologists, Atlanta, Georgia, USA

**Keywords:** adaptation, air quality, climate change, environmental health, heat, indicators, vulnerability

## Abstract

**Objective:**

To develop public health adaptation strategies and to project the impacts of climate change on human health, indicators of vulnerability and preparedness along with accurate surveillance data on climate-sensitive health outcomes are needed. We researched and developed environmental health indicators for inputs into human health vulnerability assessments for climate change and to propose public health preventative actions.

**Data sources:**

We conducted a review of the scientific literature to identify outcomes and actions that were related to climate change. Data sources included governmental and nongovernmental agencies and the published literature.

**Data extraction:**

Sources were identified and assessed for completeness, usability, and accuracy. Priority was then given to identifying longitudinal data sets that were applicable at the state and community level.

**Data synthesis:**

We present a list of surveillance indicators for practitioners and policy makers that include climate-sensitive health outcomes and environmental and vulnerability indicators, as well as mitigation, adaptation, and policy indicators of climate change.

**Conclusions:**

A review of environmental health indicators for climate change shows that data exist for many of these measures, but more evaluation of their sensitivity and usefulness is needed. Further attention is necessary to increase data quality and availability and to develop new surveillance databases, especially for climate-sensitive morbidity.

The Intergovernmental Panel on Climate Change (IPCC) projected that changes in temperature, precipitation, and other weather variables due to climate change “are likely to affect the health status of millions of people, particularly those with low adaptive capacity” (IPCC 2007) and stated that they had “very high confidence” that climate change is “currently contributing to the global burden of disease and premature deaths” (Confalonieri et al. 2007). In the United States, individual states have become the leaders in establishing carbon dioxide mitigation policies and adaptive public health programs because the establishment of a coherent U.S. mitigation policy has stalled. An example of current statewide efforts to mitigate greenhouse gases (GHGs) is the notable California legislation AB32, which mandates that greenhouse gas emissions (GHGEs) be reduced to 1990 levels by 2020 and decreased another 80% below 1990 levels by 2050 (State of California, 2006). Other states are now following California’s lead.

Unfortunately, because of previous substantial emissions, even the most optimistic reduction scenarios project that over the next few decades substantial increases in temperature and other weather changes will occur that will have large impacts on public health. For example, climate models predict that the world is expected to warm 0.5–1.0°C over the next several decades due to past emissions alone (Meehl et al. 2005; Wigley 2005). Under this scenario, increased focus on adaptive public health responses at the local level will be critical.

To develop public health adaptation strategies, evaluate their success, and project the impacts of climate change on human health, indicators of vulnerability and preparedness, along with accurate surveillance data (usually generated by state and federal environmental and health agencies) on climate-sensitive health outcomes, are urgently needed. These outcomes are important for assessing human health vulnerability to climate change (Ebi et al. 2006), for developing dose–response models (Diaz 2004), and for proposing public health preventative actions (Frumkin et al. 2008; Patz 2000). Measures of climatic fluctuations associated with climate change, such as increases in nighttime temperatures, can be used to develop early warning systems of weather patterns that can have adverse health outcomes.

The Council of State and Territorial Epidemiologists (CSTE), a U.S. professional association of public health epidemiologists, established the State Environmental Health Indicators Collaborative (SEHIC) in 2004. SEHIC comprises a group of state-level environmental health practitioners interested in developing environmental public health indicators for use within environmental health surveillance and practice. The SEHIC first focused on developing indicators for air quality, asthma, and drinking water. Last year, it established a workgroup on climate change. This article presents the initial findings of that workgroup.

## Materials and Methods

Indicators are quantitative summary measures that can be used to track changes in conditions by person, place, and time. The purpose of environmental health indicators as established by the SEHIC is to describe elements of environmental sources, hazards, exposures, health effects, and intervention and prevention activities. Indicators can be used to assess positive and negative environmental determinants of health in order to identify areas for intervention and prevention and to evaluate the outcomes of specific policies or programs aimed at improving public health. Thus, indicators serve as important communication tools for making environmental health information available to stakeholders, including environmental health practitioners, partners, policy makers, and the general public.

The SEHIC began with a comprehensive review of the scientific literature to identify outcomes and actions related to climate change that could inform recommendations about the development of a “suite” of climate change environmental health indicators. Priority was then given to identifying longitudinal data sets that were applicable at the state and community level. The workgroup recognized that indicators are needed to measure current vulnerability to climate variability and change. Indicators also are needed to track possible changes in health outcomes to determine if climate change is actually affecting their geographic range and incidence. For example, health officials are concerned that a changing climate is influencing the range of *Aedes aegypti* mosquitoes, the vector for dengue fever, because human cases of this tropical disease are increasingly found in more northern latitudes (Shope 1992). Some indicators are measures of environmental variables that can directly or indirectly affect human health, such as maximum and minimum temperature extremes. Other indicators can be used to project future health impacts based on changes in exposure, assuming exposure–response relationships remain constant over temporal and spatial scales. Based on this reasoning, we categorized four indicators: environmental, morbidity and mortality, vulnerability, and policy (i.e., implementation of adaptation and mitigation programs and activities). We identified potential data sources through web searches and by contacting data owners. Analysis of the documentation for each data source was conducted to determine data temporality, completeness, and availability. Table 1 lists the proposed indicators.

## Results

### Environmental indicators

#### GHGE/air quality

According to the U.S. Environmental Protection Agency (EPA), total U.S. GHGEs were 7,260 teragrams (Tg; millions of metric tons) of CO_2_ equivalents (Eq) in 2005, up 16% from 1990 (U.S. EPA 2008). Increased temperatures, combined with primary emissions, sunlight, and air mass stagnation events, are expected to result in increased production of ozone (O_3_) (Ebi et al. 2008; Leung and Gustafson 2005); projections for particulate matter are less consistent. The latest research indicates that O_3_ concentrations are estimated to increase 5–10% in the United States between now and the 2050s (and possibly 2.5–5% by 2030) because of climate change, if anthropogenic emissions and global background concentrations are held constant (Kinney 2008).

We recommend that GHGE and air mass stagnation events be tracked as indicators of air quality changes associated with climate variability. GHGEs are important indicators because they increase climate change and affect public health through direct effects such as heat waves, and through indirect effects such as increased growth of plant biomass that affects allergic airway disease. Air mass stagnation events, which increase O_3_ production and will increase in frequency as weather conditions favorable to heat waves increase (CCSP 2008), are another important indicator. The National Climatic Data Center (NCDC) has proposed climate impact indicators that include an air mass stagnation index. A stagnation day is defined as one with sea-level geostrophic wind < 8 m/sec, 500 millibars (mb) wind < 13 m/sec, and no precipitation (Wang and Angell 1999), and although not directly related to pollutant emissions, air stagnation days can exacerbate the effects of existing air pollution. GHGEs (CO_2_) by economic sector are easily obtainable by state from the U.S. EPA (2009), and air mass stagnation events are available by request from the NCDC (2009).

Although O_3_ levels themselves are expected to increase, it will be difficult to determine which proportion of increase of O_3_ is attributable to elevated warming from climate change and which is due to anthropogenic sources, such as population and industrial growth with concomitant emissions from mobile and stationary sources. Modeling is needed to determine the temporal increase in O_3_, after controlling for industrial and population growth and any increase in pollution controls.

#### Temperature/humidity

Along with higher temperatures, the IPCC has noted that surface specific humidity has generally increased globally after 1976 (IPCC 2007). Both high temperatures and humidity increase an individual’s risk of heat illness. Increasing temperatures directly raise body temperature, and increased humidity slows cooling of the body by decreasing sweat evaporation. Along with maximum temperatures, nighttime (minimum) temperatures are important to track for public health effects, because physiologic recovery from daytime heat is hampered if temperatures during the night do not decrease sufficiently. Vose et al. (2005) found that between 1950 and 2004, minimum global temperatures increased more rapidly than did maximum temperatures (0.204°C/decade vs. 0.141°C/decade) and resulted in a significant decrease in diurnal temperature range (−0.066°C/decade).

We recommend the following indicators to track for temperature: maximum temperature, minimum temperatures, and apparent temperature. Apparent temperature, or the use of a heat index, which combines humidity and temperature, is important in looking at mortality effects (Zanobetti and Schwartz 2008), and humidity may even become significant in drier areas of the United States as air mass moisture characteristics play a larger role during regional heat wave events. Temperature data are easily obtainable from the NCDC but require some processing effort.

#### Pollen

Increasing CO_2_ levels have been shown in laboratory and field studies to increase plant biomass and to raise the pollen production of ragweed (Rogers et al. 2006). For example, Ziska and Caulfield (2000) found that CO_2_ levels of 600 ppm projected for the middle to the late 21st century produced up to 320% more pollen in ragweed than in plants grown at preindustrial levels (280 ppm). Geographic areas with high levels of O_3_ and pollen from ragweed could cause cumulative impacts on populations; one report estimated that as many as 131 million Americans currently live in such areas (Knowlton et al. 2007).

We recommend the following indicators: pollen loads (if available or through modeling) and the presence of ragweed. Routine data for pollen loads are collected by the National Allergy Bureau (NAB). However, the spatial coverage of the monitoring stations is sparse. To obtain more complete coverage of pollen levels for the United States, either modeling or the use of satellite imagery to generate detailed land use coverage (to project the distribution of ragweed) would be necessary. However, more complete coverage via remote sensing would not provide real-time airborne pollen data, so it would be preferable to increase the number of pollen-monitoring stations.

The presence of ragweed by county is available through the U.S. Department of Agriculture (USDA) Natural Resources Conservation Service PLANTS database (USDA 2009) and the Global Biodiversity Information Facility (2009) and could serve as an interim indicator.

#### Wildfires

Increased temperatures will result in an increased frequencies of wildfires that, in turn, will elevate particulate matter levels (Kinney 2008). Large-scale wildfires and biomass burns have also been known to increase ground-level O_3_ concentrations (Jaffe et al. 2008; Val Martin et al. 2006; Thompson et al. 2001). The smoke, particulate matter, and O_3_ precursors from fires can affect local populations as well as those at long distances from the fire’s origin (Colarco et al. 2004; DeBell et al. 2004). Besides increased temperatures, higher CO_2_ levels are likely to contribute to higher plant biomass (“CO_2_ fertilization”), which could increase the potential for wildfires (Arizona Cooperative Extension 2006).

Wildfire risk is likely to vary regionally with projected increases in the frequency, severity, distribution, and duration in the U.S. Southeast, the Intermountain West, and West (Committee on Environment and Natural Resources 2008). For example, in California, early snowpack melt due to warmer spring temperatures results in a longer and drier wild-fire season, which is exacerbated by drought conditions. Analysis of wildfires trends has shown that wildfire activity in the western United States became more prevalent in the mid-1980s, with greater frequency and duration, and longer wildfire seasons (Westerling et al. 2006). As warmer and earlier springs are projected with climate change, more wildfires may result in significant loss of carbon sink, along with increased levels of CO_2_.

Data from the National Interagency Fire Center (2009) can be used to monitor national wildfire trends. Recommended indicators include examining the frequency, severity, distribution, and duration of wildfires. Suggested measures include the annual area burned and the average yearly increase in the proportion of acres burned. More research is needed to determine which is the appropriate baseline year for analysis, but it most likely would be limited to the earliest year of data available (Table 1).

#### Drought

Drought indicators should be monitored by public health officials because drought is associated with degraded water quality and quantity, waterborne disease, and food safety, among other concerns (Georgia Water Advisory Group 2007).

There is no single indicator for drought. Several indices are available, including percent of normal, the standardized precipitation index (SPI), the Palmer Drought Severity Index, and the surface water supply index (SWSI) [National Drought Mitigation Center (NDMC) 2006]. The NDMC uses the SPI because it can project emerging droughts sooner than other indices. It is recommended that the SWSI be used in western states, where water quantity and quality are dependent on snow pack levels. Therefore, we recommend that the SPI and SWSI be used as climate change drought measures. Several web-based tools exist for monitoring drought and its effects, such as the NDMC’s Drought Impact Reporter (2009), which monitors drought effects on agriculture, water/energy, environment, fire, and social factors.

To assess the impact of drought on human populations, Falkenmark et al. (1989) used water scarcity (water supply < 500 m^3^/person) and water stress (water supply < 1,000 m^3^/person) as indicators.

#### Harmful algae blooms

A worldwide increase in cyanobacterial (blue-green algae) sources has been observed in both coastal and freshwaters (Hallegraeff 1993; Moore et. al. 2008). These harmful algae blooms (HABs), which produce nerve and liver toxins, are longer in duration, of greater intensity, and are suspected of being tied both to increased temperatures due to climate change and nutrient runoff. Exposure to marine toxins has resulted in death and poisonings of California sea lions and Florida alligators. Human exposure is of concern through both drinking water contamination and recreational exposure. Human exposure to HABs can cause eye and skin irritation, vomiting and stomach cramps, diarrhea, fever, headache, pains in muscles and joints, and weakness. Chronic exposure in drinking water supplies is suspected to have links with liver damage and cancer (Svircev et al. 2009).

Potential indicators include shellfish poisoning and blue-green algae and red tide outbreaks. Outbreaks of shellfish poisonings and red tides in the ocean could be monitored, along with blue-green algae outbreaks in freshwater. Shellfish poisoning outbreaks in humans, however, are typically underreported and often misdiagnosed. The National Oceanic and Atmospheric Administration (NOAA) maintains a Harmful Algae Bloom Forecasting System (NOAA 2009a) that tracks the location, extent, and potential movement of HABs in the Gulf of Mexico. Monitoring freshwater HAB outbreaks has focused mainly on the Great Lakes, although some states monitor outbreaks, along with local beach and surface water closings, for shellfish contamination or red tides.

### Morbidity and mortality indicators

#### Mortality and morbidity from extreme heat

The IPCC projects with “virtual certainty” that climate change will cause more frequent, more intense, and longer heat waves. It also notes with “medium confidence” that the number of heat wave deaths will increase (medium confidence arose because of uncertainty regarding physiologic and societal adaptation) (Confalonieri et al. 2007). All heat wave deaths are preventable. They are, however, difficult to identify because few deaths are recorded as heat-related during a heat wave compared with retrospective analyses (Shen et al. 1998). For similar reasons, heat illness is rarely listed as a primary cause of death on death certificates for deaths that occur in hospitals or emergency rooms (ERs). For instance, heart failure or respiratory conditions may be listed as the primary cause, with heat illness as a contributing factor. Until recently, little research and data have been available on morbidity effects from hyperthermia. Data recently became available on ER visits for analysis in some states. For example, Knowlton et al. (2009) found that 16,166 excess ER visits and 1,182 excess hospitalizations occurred in 2006 during the 2-week heat wave in California.

We recommend indicators for mortality and morbidity from extreme heat to include excess mortality and morbidity. Mortality data at a statewide level are available from CDC’s National Center for Health Statistics (NCHS), which defines a death as heat-related when heat is the underlying or contributing cause of death. To document the full impact of a heat event on mortality, it is important to calculate the excess mortality associated with an event because some deaths would have occurred regardless of the weather conditions. Excess mortality can be calculated by comparing the number of deaths during an extreme temperature event with those during a reference period that has been matched by day of the week and other potentially confounding factors, or by using a time-series approach. For example, during the California 2006 heat wave, 655 excess all-cause deaths occurred, a statistically significant increase of 6% [RR = 1.06; 95% confidence interval (CI), 1.03–1.09] (Hoshiko et al. 2009). During the same time period in 2007, only 140 deaths were reported on coroners’ reports (Trent 2007).

On a national scale, Medicare and Medicaid data are available to analyze the morbidity impact of heat waves on poor and elderly populations. Other vulnerable populations, such as non-Medicaid children, are not covered by these data. The Agency for Healthcare Research and Quality (AHRQ) provides access to community hospital inpatient and ER data through its Healthcare Cost and Utilization Project (HCUP). Data from some participating states are publicly available through HCUPnet, an online access point for HCUP (AHRQ 2009). Through HCUPnet, hospitalization data are available for 30 states, but coverage varies between 1997 and 2006 (most recent year available). Just 7 states provide access to their ER data through HCUPnet and are currently available only for 2005.

In addition, it is important to track the presence and effectiveness of heat wave early warning systems, because they are critical determinants of the extent of mortality during a heat wave. Further, development of such systems and local heat response emergency plans will be critical for adaptation for chronic heat stress (McGeehin and Mirabelli 2001).

#### Extreme weather event injuries and mortality

Increases in heavy precipitation related to climate change and earlier regional snow melt and temperature variability raise risks of flooding and related community displacement and injuries. Strong Atlantic hurricanes are projected to increase in intensity, and strong cold weather storms are expected to become more frequent (CCSP 2008). After Hurricane Katrina, approximately 17,500 case reports were filed in hospitals and acute care clinics in the greater New Orleans area, with 51.6% infectious and noninfectious disease related and 26.2% injury related [Centers for Disease Control and Prevention (CDC) 2006a]. There were 971 deaths from Katrina in Louisiana, with drowning, injury and trauma, and heart conditions the leading causes of death (Eavey and Ratard 2008). Other post-Katrina events exacerbated pre-existing chronic conditions from population displacement, mental health issues, and infectious disease (CDC 2006b). Floods are the most frequent natural disaster in the United States and, before Katrina, accounted for 40% of all natural disaster damage and injury (Greenough et al. 2001). A review of National Weather Service (NWS) flash-flood–related deaths in the United States from 1969 to 1981 found that 1,185 deaths occurred during 32 flash floods (French et al. 1983).

We recommend mortality from flooding and storms for indicators in this area. These data are available from the Emergency Events Database (EM-DAT) at the country level from the Centre for Research in the Epidemiology of Disasters (CRED) in Belgium (CRED 2009). Data are compiled from various sources, including United Nations agencies, governments, and the International Red Cross. In the United States, the NWS and NOAA report state-level summary statistics on injuries and mortality resulting from extreme weather events in their storm events data reports. Health data are likely to be severely underreported because these agencies rely heavily on newspaper and other media reports for information. We examined national death files (CDC 2009c) for injury codes citing “victim of flooding” (X32) and found only 12 deaths reported nationwide in 2005. This finding suggests that this code is not used routinely on death certificates and that other diagnoses are used as primary cause of death in these cases. At this time, no domestic surveillance database exists for deaths and injuries for extreme weather events.

#### Environmental infectious disease

Climate change may affect the geographic range and incidence of several environmental infectious diseases, including West Nile encephalitis, Lyme disease, coccidioidomycosis (“valley fever”), dengue fever, and human hantavirus cardiopulmonary syndrome (HCPS). Cases of dengue fever have been found at the U.S./Mexico border. The southern and southeastern United States are considered at risk for the illness because of the presence of the mosquito vector *Aedes aegypti* and the emerging vector *A. albopictus*.

Recommended indicators include human cases of West Nile virus (WNV; along with the number of positive tests for mosquito and sentinel species), Lyme disease, dengue fever, coccidioidomycosis, and HCPS. Surveillance data for human cases of environmental infectious diseases and disease vectors and reservoirs are routinely collected by state programs and reported to CDC’s ArboNET surveillance system. Several environmental infectious diseases have been cited in the literature as likely to undergo a change in the quantity of human disease cases, or in the geographic range of vectors or reservoirs as a result of climate change. Human cases of WNV have been mapped by the U.S. Geological Survey (USGS) using data submitted to the ArboNET program (also available are St. Louis encephalitis, western equine encephalitis, eastern equine encephalitis, La Crosse encephalitis, and Powassan virus for various years) (USGS 2009). Historical data for WNV are available back to 1999. Trends in human cases of WNV disease vary by region. For example, California had a total of 380 WNV human symptomatic cases in 2007, 278 in 2006, and 880 in 2005 [California Department of Public Health (CDPH) 2009]. Maps and data are also maintained of positive test results for WNV in mosquito pools and in sentinel species (both 2001–2006).

Many state health departments, along with the CDC Division of Vector-Borne Infectious Diseases, conduct surveillance for Lyme disease, which is found primarily in the northeastern United States. The incidence of this condition has increased considerably from 1992 to 2006, although part of the increase may be due to factors such as increased surveillance (CDC 2008). Data from studies on the range of the established populations of the Lyme disease vectors *Ixodes scapularis* and *I. pacificus* are limited (Brownstein et al. 2003; Dennis et al. 1998). Maps of dengue fever outbreaks can be found at the CDC Division of Vector-Borne Infectious Diseases website (CDC 2009d). Although rare, HCPS has been detected in 30 states in the United States. The CDC Division of Vector-Borne Infectious Diseases maintains counts of the illness by state (CDC 2009f). Coccidioidomycosis occurs primarily in the southwestern United States; surveillance data are available from the California and Arizona state health departments.

#### Respiratory and allergic disease and mortality related to air quality and pollens

Relatively few studies have attempted to document increases in mortality and other health impacts due to climate change increases in O_3_ or other pollutants. Ebi and McGregor (2008) reviewed studies analyzing impacts of climate change on air quality and health and concluded that the studies generally indicate that O_3_ levels will increase, especially in high-income countries, resulting in increased morbidity and mortality. Accounting for climate change and O_3_ precursor emissions and population growth, Knowlton et al. (2004) estimated a median 4.5% increase in O_3_-related acute mortality across 31 New York metropolitan area counties by the 2050s. Estimating these impacts and developing indicators depends upon progress in accurate regional-scale air models of climate change impacts on O_3_. In a study with a larger scope, Bell et al. (2007) estimated the impacts of projected increases of O_3_ on total mortality in 50 U.S. cities by 2050. Holding the effects of anthropogenic emissions of O_3_ precursors constant, they found that O_3_ levels (daily 1-hr maxima) were projected to increase 4.8 ppb, resulting in a 0.11–0.27% increase in daily total mortality.

### Population vulnerability indicators

In the analysis of population vulnerability to climate change, it is important to recognize that specific populations will be vulnerable to different climate-sensitive outcomes. For example, those with preexisting asthma and chronic obstructive pulmonary disease will be particularly vulnerable to temperature-related effects of O_3_ (Gamble et al. 2008). Children have also been identified as especially susceptible to many of the effects of climate change, such as flooding, heat, and air pollution (Perera 2008). Vulnerability can be assessed by not only documenting baseline exposures, but also by taking into account population sensitivities, the capacity to adapt, and how individuals and society respond to climate threats (Gamble et al. 2008).

Vulnerable populations are persons who are independent on a daily basis, but during and after an emergency may require assistance to meet their basic needs. This includes, but is not limited to, persons with preexisting chronic diseases, individuals with disabilities (physical or mental), the elderly, low-income populations, and children. Any change in their daily routine may become a stressor. Population vulnerability indicators are important for public health and emergency response officials to target susceptible communities for prevention and intervention activities.

#### Heat vulnerability/drought

Populations that have been found to have high vulnerability to heat mortality and morbidity include the socially isolated, children, the poor, and the elderly. Reid et al. (2008) conducted a principal component analysis to construct an index of community heat vulnerability at the census tract level, which combined vulnerability factors from the U.S. Census with air conditioning data from the American Housing Survey and comorbidity data from the Behavioral Risk Factor Surveillance System (BRFSS). This approach could be coupled with heat exposure surfaces to show the intersection between exposure and vulnerability. For example, in urban areas, satellite imagery that can document urban heat islands and temperatures at a neighborhood scale has been linked with data on social vulnerabilities (Vescovi et al. 2005; Wilhelmi et al. 2004). Additionally, data on acute health events such as address-level ambulance response calls for heat stress could be used for map validation.

Other vulnerable populations affected by drought include dialysis patients, the elderly, pregnant and nursing women, infants, immunocompromised individuals (e.g., chemotherapy and AIDS patients), and persons with preexisting health conditions, such as hypertension and diabetes.

Proposed indicators that can be used to map vulnerabilities for heat mortality and drought are available from the U.S. Census and include population distributions of elderly persons living alone, poverty status, children, infants, and individuals with disabilities.

#### Flooding

As with extreme weather events in general, populations vulnerable to the impacts of flooding include the elderly and the poor (Ahern et al. 2005; Bernard and Ebi 2001; Ebi et al. 2006; Gabe et al. 2005). In addition, people who live in areas that have experienced little or no flooding in the past are often more vulnerable to health impacts because they are less prepared and less experienced in dealing with floods (Barredo et al. 2007).

Other groups with increased vulnerability to climate change include infants, immunocompromised persons, those with chronic diseases or receiving drug treatment, and the obese (Ebi et al. 2006). Researchers have also noted that immobility due to lack of transportation (Colten 2006; U.S. Government Accountability Office 2006) or disability (Gabe et al. 2005) is associated with greater vulnerability to climate change impacts and that these same factors are related to poverty.

Recommended indicators include the percentage of elderly, those in poverty, infants, and the disabled living in 100- and 500-year flood zones. Paper floodplain maps have been generated by the Federal Emergency Management Agency (FEMA) for zoning and insurance purposes. FEMA is currently modernizing its mapping process, the Digital Flood Insurance Rate Maps (DFIRMs). More importantly, FEMA is updating the flood risk zones on the DFIRMs, which were found to be inadequate in projecting flood risk, such as the aftermath of Hurricane Floyd in 1999. To identify populations vulnerable to displacement from flooding, census data can be coupled with digital flood zone maps to identify the number and percentage of populations living in 100- and 500-year flood zones. These maps could be further refined to identify the percentage of elderly persons, the percentage of persons living in poverty, and those with comorbidities and other restrictions in these areas.

#### Sea-level rise

A recent study has projected that the mean sea level along the California coast will rise from 1.0 to 1.4 m by the year 2100 under medium to medium-high emissions scenarios (Cayan et al. 2009). Coastal and inland communities near sea level will be subject to infrastructure loss, both financial and social.

**Figure 1:**
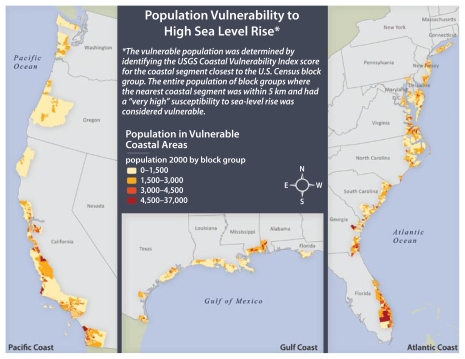
Population by U.S. county within 5 km of the coast with “very high” vulnerability to sea level rise. Data from the USGS (2000) and U.S. Census (2000).

The USGS has developed an index of coastal vulnerability to future sea-level rise, which incorporates tidal range, wave height, coastal slope, shoreline erosion rates, geomorphology, and historical rates of sea-level rise (Thieler 2000). Coastal vulnerability is ranked from low to very high. We used the coastal vulnerability index and population data and boundaries for coastal census block groups for the contiguous United States to create a measure that provides a general indication of the population living in close proximity to high-risk areas. Figure 1 shows the population by county within 5 km of coast with “very high” vulnerability to sea level rise. Areas in California and Florida show the greatest populations at risk.

### Mitigation, adaptation, and policy indicators

As mentioned previously, mitigation has been the primary focus of state climate change efforts in the United States. Adaptation is just as important as mitigation to reduce short-term and longer term health risks. However, limited attention has been paid to public health adaptation to climate change until recently. Adaptation indicators are needed to measure the status of public health efforts to avoid, prepare for, and effectively respond to the risks of climate change.

Data on mitigation indicators are available from federal sources. Proposed mitigation indicators are energy efficiency levels, use of renewable energies, and vehicle miles traveled. For example, the Department of Energy’s Energy Information Administration (EIA) collects information on energy consumption in U.S. households by census region and type of housing unit (U.S. Department of Energy 2009). The EIA also tracks renewable energy use and the number of vehicle miles traveled by state.

Data on adaptation indicators are sparse and most likely will need to be collected by public institutions or other organizations using surveys. Proposed indicators include community access to cooling centers during heat waves (and transportation to the centers); heat wave early warning systems; municipal heat island mitigation plans; surveillance systems per state that collect data on the human health effects of climate change; and a public health workforce trained in climate change research, surveillance, or adaptation. A city or region may also set up an adaptation climate change task force that includes a representative from the health sector.

Heat warnings and alerts are issued by the NWS and by early warning systems in various cities. A list of heat alerts and warnings by jurisdiction is available in the NWS’s storm event database (NWS 2009), but data completeness and accuracy are questionable. The Storm Events data are compiled from the NWS but also may include unverified data from sources outside the service. Further, the focus of many of these systems is on forecasting weather conditions that can adversely affect health, with limited focus on the public health response activities (Bernard and McGeehin 2004). Recent surveys show that although awareness of heat wave warnings is high, less than 50% of vulnerable populations change their behavior in response to a warning (Abrahamson et al. 2008; Sheridan 2007). Individuals at greatest risk, such as elderly persons living alone and lacking social contacts, often lack the resources to protect themselves from the health effects of heat.

Finally, data on some proposed policy indicators are available, which include the number of cities or municipalities covered by the Kyoto protocol and the number of states and local jurisdictions participating in climate change initiatives, such as climate registries or the U.S. Mayors’ Climate Protection Agreement. Data are available from the Mayor’s Climate Protection Center (2009) and the International Council for Local Environmental Initiatives (ICLEI 2009).

## Discussion

Ebi et al. (2008) assessed the potential health impacts of climate change for U.S. populations and concluded that climate change poses a health risk. They concluded that

- It is very likely that heat-related illnesses and deaths will increase over coming decades.
- A growing body of evidence indicates that O_3_ concentrations are more likely to increase than to decrease in the United States as a result of climate change, if one assumes that precursor emissions are held constant. An increase in O_3_ could cause or exacerbate heart and lung diseases.
- Because it is not yet possible to project changes in future extreme climate change events, researchers cannot estimate the exact health impacts that may result from these events. However, potentially serious health consequences do exist when such events occur. Health risks associated with extreme events are likely to increase because of an increasing population and the degree to which people are physically or financially constrained or uninformed about their ability to prepare for and respond to extreme weather events.
- The very young and old, the poor, those with health problems and disabilities, and certain occupational groups are at greater risk.
- Health burdens related to climate change will vary by region.

In order to evaluate these impacts, we have presented a recommended list of surveillance indicators that include not only climate- sensitive health outcomes but also environmental, population vulnerability, and mitigation, adaptation, and policy indicators of climate change. Besides evaluating the health impact of climate change, developing these indicators is also vital for program evaluation, health service planning, and communication. For example, one issue that requires attention is to refine the spatial scale of the recommended indicators, which currently vary widely. In fact, some indicators cannot be used at local geographic scales. Until finer scale surveillance methods can be implemented, the modeling of the indicators should be downscaled to the local level. For example, O_3_ modeling should be done at smaller spatial scales to estimate impacts on local areas. However, finer resolution of health outcome data, such as morbidity and mortality, may involve confidentiality restrictions and may prevent data sharing unless spatial smoothing techniques are employed (Roberts et al. 2008).

Further indicator development will be hampered by sensitivity and data quality and availability issues. Sensitivity will not be uniform for all indicators. For example, although an increase in heat waves is projected, it is not a foregone conclusion that morbidity and mortality will increase to the same degree in all locations. Health impacts from heat waves would be affected by local preparedness infrastructure, personal health behaviors, acclimatization, and the built environment. Public health agencies and partner organizations can affect the likelihood of health effects from occurring by developing and promoting emergency heat warning systems, improving heat risk communication and education, and working with planners to minimize heat island effects.

Further evaluation, validation, and research are needed to determine the health effects of proposed indicators such as air mass stagnation events and HABs. Additional investigations are needed to determine the role of warming on plant biomass and ragweed and the implications of increased pollen, allergies, and asthma (D’Amato and Cecchi 2008; Shea et al. 2008) and the effects of increased O_3_ on respiratory conditions (Bell et al. 2007).

Data gaps are especially critical for some environmental and population vulnerability indicators. No national surveillance data set is available to analyze hyperthermia impacts on morbidity for all U.S. populations. For example, BioSense (CDC 2009b), the national system to access real-time hospitalization data, has incomplete coverage and is inadequate to conduct timely surveillance on health impacts from such events as wildfires.

No domestic surveillance database exists for deaths and injuries for extreme weather events. The NWS, state health departments, and CDC shuld work together to form an accurate system for recording these cases on a state-by-state basis in the United States. For environmental infectious diseases, a surveillance system needs to be developed for ongoing examination of the range and distribution of Lyme disease and dengue fever vectors.

For population vulnerability indicators, the greatest needs include modernizing and implementing FEMA’s project and identifying and communicating information to those at high risk of heat morbidity and mortality. The paucity of data on adaptation and policy indicators is evident, and we encourage organizations such as the Association of State, Territorial and Health Officials, CSTE, and other organizations to undertake surveys to collect this information. In addition, the NWS should work with health organizations to standardize heat alerts and warnings and benchmark them to public health outcomes. These warnings need to be coupled with public health responses.

In conclusion, a review of proposed environmental health indicators for climate change in the United States shows that data exist for many environmental and health measures, but more research is needed to evaluate the sensitivity and usefulness of these measures. Further attention is necessary to increase data quality and availability and to develop new environmental monitoring and surveillance databases, especially for climate-sensitive morbidity. Maintaining the public health infrastructure by adequately funding environmental and chronic disease surveillance systems and a well-trained public health workforce are critical.

## Figures and Tables

**Table 1:** Proposed environmental health indicators for climate change.

Indicator	Data source	Years available	Limitations
Environmental indicators			
GHGEs	U.S. EPA 2009	1990–2005	Lists emissions from fossil fuels only
Stagnation air mass events	NOAA 2009b	1948–present	Not applicable
O_3_ estimates due to climate change	Bell et al. 2007; Ebi and McGregor 2008; Knowlton et al. 2004; Shea et al. 2008; Thompson et al. 2001; Val Martin et al. 2006	NA	Based on modeling
Maximum and minimum temperatures, heat index	NCDC 2009a	1900–present	Temperature monitors not always present in population centers
Increase in heat alerts/warnings	NOAA 2009c; NWS 2009	1993–present	Data completeness and accuracy questionable
Pollen counts, ragweed presence	Global Biodiversity Information Facility 2009; NAB 2009; SDI Health LLC 2009; USDA 2009	Varies by source	Limited number of pollen-monitoring stations (only 78 report to the National Allergy Bureau)
Frequency, severity, distribution, and duration of wildfires	National Interagency Fire Center 2009	1960–2007	Not applicable
Droughts: SPI, SWSI	NDMC 2006	1901–present	Need to analyze precipitation data available from NCDC
HABs: human shellfish poisonings, HAB outbreak monitoring in freshwater and ocean waters	Specific states: NOAA 2009a	Ongoing	Shellfish poisonings are underreported and misdiagnosed; there is limited monitoring of freshwater HAB outbreaks

Morbidity and mortality indicators			
Excess mortality due to extreme heat	CDC 2009g	1968–2005	Not applicable
Excess morbidity due to extreme heat	CDC 2009b; CMS 2009	AHRQ HCUPnet hospitalization data availability vary for 30 states between 1997 and 2006, and ED data are available for seven states for 2005	Coverage only for low-income and Medicaid files, and elderly in Medicare; AHRQ files not complete for all states; BioSense has limited coverage of participating facilities
No. of injuries/mortality from extreme weather events	CDC 2009g; CRED 2009; NCDC 2009b	CRED, 1900–present; NCDC, 1993–present; NCHS, 1968–2005	Underreporting and inconsistencies in reporting in U.S. data sources
Human cases of environmental infectious disease/positive test results in reservoirs/sentinels/vectors	CDC 2009e	West Nile virus, 1999–present; Lyme disease, 1992–present	Limited data on range of vector for Lyme disease
Respiratory/allergic disease and mortality related to increased air pollution and pollens	Bell et al. 2007; Confalonieri et al. 2007; D’Amato and Cecchi 2008; Ebi and McGregor 2008; Ebi et al. 2006; Gamble et al. 2008; Kinney 2008; Knowlton et al. 2004; Shea et al. 2008	NA	Based on modeling

Vulnerability indicators			
Elderly living alone, poverty status, children, infants, and individuals with disabilities	CDC 2009a	1960–2000 (U.S. Census); 1984–present (BRFSS)	Needs to be coupled with heat exposure data
Flooding vulnerability (elderly, those in poverty, infants, and disabled living in 100- and 500-year flood zones)	FEMA 2009; U.S. Census 2009	1960–2000 (U.S. Census)	Flood plain maps are undergoing digital revisions
Sea level rise vulnerability (population by county within 5 km of coast with “very high” vulnerability to sea level rise)	USGS 2000	NA	NA

Mitigation indicators			
Energy efficiencies	Department of Energy 2009	1978–2001	NA
Use of renewable energy	Department of Energy 2009	2002–2006	
No. of vehicle miles traveled	Department of Energy 2009	1983–1993	

Adaptation indicators			
Access to cooling centers	Surveys (no surveys are currently available)	NA	NA
No. of heat wave early warning systems	Surveys	NA	
No. of municipal heat island mitigation plans	Surveys	NA	
No. of health surveillance systems related to climate change	Surveys	NA	NA
Public health workforce available/trained in climate change research/surveillance/adaptation	Surveys	NA	NA

Policy indicators			
No. of cities/municipalities covered by Kyoto protocol	U.S. Conference of Mayors Climate Protection Center 2009	NA	NA
No. of states/cities participating in climate change initiatives	ICLEI 2009	NA	NA

Abbreviations: AHRQ, Agency for Healthcare Research and Quality; BRFSS, Behavioral Risk Factor Surveillance System; CRED, Centre for Research in the Epidemiology of Disasters; FEMA, Federal Emergency Management Agency; ICLEI, International Council for Local Environmental Initiatives; NA, not applicable; SPI; standardized precipitation index; SWSI, surface water supply index; USGS, U.S. Geological Survey. Data from the State Environmental Health Indicators Collaborative, Council for State and Territorial Epidemiologists (unpublished data).
